# Association between nonalcoholic fatty liver disease and increased glucose-to-albumin ratio in adults without diabetes

**DOI:** 10.3389/fendo.2023.1287916

**Published:** 2024-01-08

**Authors:** Shuai Wang, Xiaohong Lin, Chuchen Zhu, Yuqi Dong, Yiwen Guo, Zhonghao Xie, Xiaoshun He, Weiqiang Ju, Maogen Chen

**Affiliations:** ^1^ Organ Transplant Center, First Affiliated Hospital of Sun Yat-sen University, Guangzhou, China; ^2^ Guangdong Provincial Key Laboratory of Organ Donation and Transplant Immunology, Guangzhou, China; ^3^ Guangdong Provincial International Cooperation Base of Science and Technology (Organ Transplantation), Guangzhou, China; ^4^ Department of Breast and Thyroid Surgery, Eastern Hospital of the First Affiliated Hospital of Sun Yat-sen University, Guangzhou, China

**Keywords:** nonalcoholic fatty liver disease, diabetes, advanced hepatic fibrosis, donor liver, glucose-albumin ratio

## Abstract

**Background:**

Nonalcoholic fatty liver disease (NAFLD) affects approximately 30% of individuals globally. Both serum glucose and albumin were demonstrated to be potential markers for the development of NAFLD. We hypothesized that the risk of NAFLD may be proportional to the glucose-to-albumin ratio (GAR).

**Methods:**

Based on information from the National Health and Nutrition Examination Survey (NHANES) 1999–2018, it was determined that GAR was associated with an increased risk of NAFLD and liver fibrosis utilizing weighted multivariable logistic regression. Participants with a fatty liver index (FLI) over 60 were identified with NAFLD, and those with an NAFLD fibrosis score (NFS) >0.676 with evidence of NAFLD were labeled with advanced hepatic fibrosis (AHF). The liver biopsy was utilized to verify the relationship between GAR and FLD in our center cohort. Mendelian randomization analysis investigated the genetic relationship between GAR and NAFLD.

**Results:**

Of 15,534 eligible participants, 36.4% of participants were identified as NAFLD without AHF. GAR was positively correlated with the probability of NAFLD following full adjustment for possible variables (OR = 1.53, 95% CI: 1.39–1.67). It was confirmed that patients with NAFLD and AHF had an inferior prognosis. The relationship between GAR and NFS was favorable (R = 0.46, P< 0.0001), and NAFLD patients with a higher GAR tended to develop poor survival. In our center cohort, the association between GAR and NAFLD was verified.

**Conclusion:**

Among participants without diabetes, greater GAR was linked to higher risks of NAFLD. In addition, NAFLD patients with higher GAR tended to develop liver fibrosis and adverse outcomes.

## 1 Introduction

Nonalcoholic fatty liver disease (NAFLD) excludes viral, alcoholic, drug, and other secondary liver illnesses and is distinguished by abnormal fat buildup for more than 5% of the liver volume (1). A systematic review in 2023 presented that approximately 30% of people worldwide were affected by NAFLD (1). NAFLD has become more and more prevalent, with an increasing rate of 1% per year (2). Additionally, liver steatosis is widespread in the donor organ pool due to the global increase in NAFLD incidence (3). Therefore, the research on the risk and prognostic factors for NAFLD is worth shedding light on.

While various liver examination methods were invented during the past few years, liver biopsy still was the gold standard (4). There was an urgent need for authentic noninvasive approaches for identification and risk stratification of NAFLD due to the significant drawbacks that liver biopsy presents.

NAFLD is demonstrated to be associated with several metabolic syndromes such as abdominal obesity, dyslipidemia, hyperglycemia, and hypertension (5). A consensus published in 2022 endorses the name metabolic (dysfunction)-associated fatty liver disease (MAFLD) due to the underlying pathogenesis of NAFLD (6). Numerous studies insist that serum glucose contributes to the development and progress of NAFLD. More specifically, 24-h glucose concentrations measured by the area under the curve (AUC) value elevate the level of hepatic *de novo* lipogenesis (DNL) in NAFLD patients (7). Furthermore, the relationship between glucose and NAFLD is verified both *in vivo* and *in vitro*. The addition of glucose leads to lipid accumulation in primary human hepatocytes (8). High glucose induces apoptosis in HepG2 cells through increasing oxidative stress (9). Serum glucose fluctuation induced by intermittent glucose injection contributes to apoptosis, inflammation, fibrosis, and oxidative stress, even mitochondrial dysfunction in male C57BL/6J mice (10). Therefore, more and more research investigates the role of glucose in the early identification of NAFLD. The factor reflecting glucose and lipid metabolism, the triglyceride glucose (TyG) index, assisted with the early identification of NAFLD, and even liver fibrosis (11–13). However, there are no other combined indexes including glucose at present for NAFLD patients. Similarly, a new index, the glucose-to-albumin ratio (GAR) is illustrated in a recently published cross-sectional research (14). Albumin is produced by the liver and takes a role of antioxidation and anti-inflammation (15). There are reduced albumin synthesis and aberrant structure and function of albumin in the serum of liver cirrhosis patients (16, 17). Several studies have demonstrated that hypoalbuminemia is related to a poorer prognosis for NAFLD patients (18, 19). Given the distinct predictive significance of albumin and serum glucose discussed above, GAR seems to be a more effective factor for the identification and risk classification of NAFLD.

To examine a clinically available monitoring indicator of NAFLD, we employed the 1999–2018 National Health and Nutrition Examination Survey (NHANES) cohort and our center cohort for investigating and assessing the connection of GAR with NAFLD risk in a population without diabetes.

## 2 Methods

### 2.1 Subjects

To determine the health and nutritional status of adults and children in the United States, the NHANES was established. This initiative compiles data pertaining to social, nutritional, and health-related issues. Furthermore, a sophisticated, stratified, multistage, and probability cluster-designed sampling process is carried out in the data collection of NHANES, which means that the weighted analysis is indispensable for this research. Data for NHANES 1999-2018 containing 101,316 participants were included in this study. Then, we included all of the adult participants without diabetes (n = 52,253), who were characterized by age over 18 years and completed demographic information (age, gender, and race). Participants were excluded if they: (1) omitted details necessary for the definitions of NAFLD or advanced hepatic fibrosis (AHF); (2) had alcoholic fatty liver (more than three standard drinks per day for men or more than two standard drinks per day for women); (3) were diagnosed with viral hepatitis (hepatitis B surface antigen or hepatitis C virus RNA positivity); (4) had taken prescription drugs with the potential to impact hepatic steatosis in the last month; (5) had self-reported cancer status; (6) had an irregular energy intake of 4,200 kcal per day or an unsatisfactory dietary recall status. Finally, a total of 15,534 subjects were acquired as eligible participants.

**Figure 1:**
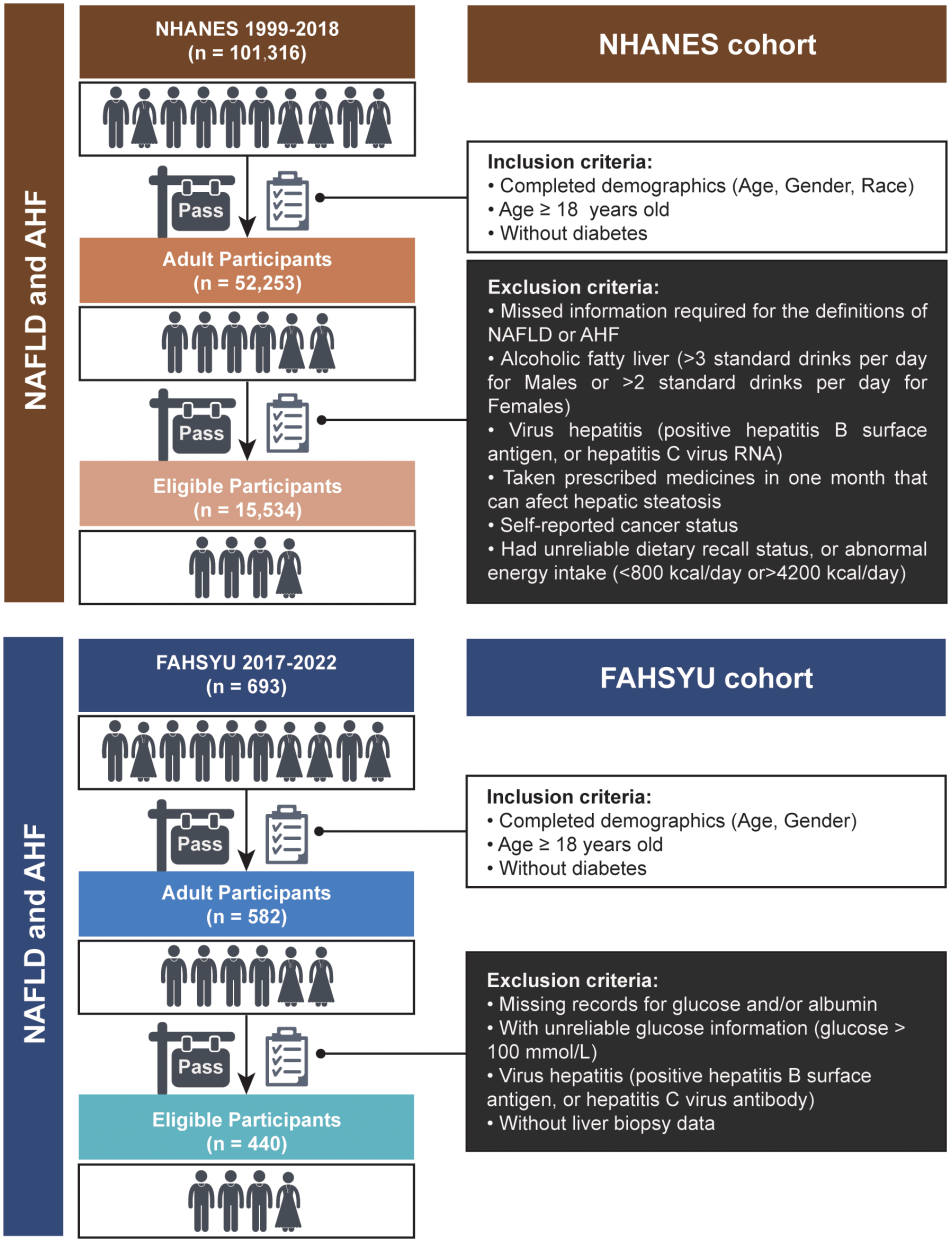
Flowchart for the sample selection in NHANES and FAHSYU cohorts. NAFLD, nonalcoholic fatty liver disease; AHF, advanced hepatic fibrosis; NHANES, National Health and Nutrition Examination Survey; FAHSYU, First Affiliated Hospital of Sun Yat-sen University.

From 1 January 2017 to 1 September 2022, 693 donors in our center (Organ Transplant Center, First Affiliated Hospital of Sun Yat-sen University; FAHSYU) received liver transplantation, and corresponding clinical characteristics and biopsy profiles were preserved. In this study, we included all of the adult donors without diabetes (n = 582) who were aged over 18 years and possessed completed demographics (age and gender). Then, the donors were excluded if they: (1) had missing records for glucose and/or albumin; (2) had unreliable glucose information (glucose >100 mmol/L); (3) were identified as having the hepatitis virus (positive screening for the hepatitis B surface antigen or the hepatitis C viral antibody); (4) possessed liver biopsy data. Finally, a total of 440 donors were defined as the validation cohort in our research (**Figure 1**). In addition, the cause of death for all of the donors was listed in **Supplementary Table S1**.

### 2.2 Serum glucose and albumin measurements

The NHANES data were classified into five sections: demographics, dietary, examination, laboratory, and questionnaire data. The frozen serum samples were applied to assess serum glucose and albumin. After that, a multichannel Hitachi Model 917 analyzer (Roche Diagnostics, Indianapolis, IN, USA) was employed to do their examination. On the official website, you can access more specific information on how to collect samples and other methodological techniques (https://wwwn.cdc.gov/nchs/nhanes/AnalyticGuidelines.aspx). The GAR was calculated according to the formula:


$$\frac{Serum\;Glucose\;\left(mmol/L\right)}{Serum\;Albumin\;\left(g/L\right)}\times 10^{2}$$

### 2.3 Measurements of other covariates

The demographic data contain age, gender, race, education level, and family poverty income ratio (PIR), which was acquired from the household interview. A qualified examiner at the mobile examination center (MEC) tracked each candidate’s weight, height, waist circumference (WC), systolic blood pressure (SBP), and diastolic blood pressure (DBP). Body mass index (BMI) was defined as weight (in kg) divided by height squared (in m^2^). The NHANES computer-assisted dietary interview (CADI) system was utilized to obtain detailed dietary intake information, such as energy, protein, carbohydrate, and total fat. Serum alanine aminotransferase (ALT), aspartate aminotransferase (AST), alkaline phosphatase (ALP), γ-glutamyl transferase (GGT), and bilirubin were tested similarly to serum glucose and albumin with a Hitachi Model 704 multichannel analyzer (Boehringer Mannheim Diagnostics, Indianapolis, IN, USA). The completed blood cell count was measured by the Beckman Coulter MAXM instrument, which contains white blood cells, lymphocytes, neutrophils, eosinophils, basophils, red blood cells, and platelets. To determine the amounts of triglycerides, total cholesterol, high-density lipoprotein (HDL) cholesterol, and low-density lipoprotein (LDL) cholesterol, frozen blood samples were delivered to the Johns Hopkins University Lipoprotein Analytical Laboratory. The glycated hemoglobin (HbA1c) was examined by the University of Missouri-Columbia for analysis. The profiles about whether to take lipid-lowering medications, glucose-lowering therapies, or antihypertensive medications were acquired through a questionnaire. The NHANES Laboratory/Medical Technologists Procedures Manual (LPM) contains comprehensive guidelines for collecting and processing specimens.

Taking lipid-lowering drugs or having a triglyceride level greater than 150 mg/dL, an LDL cholesterol level higher than 140 mg/dL, or an HDL cholesterol level less than 40 mg/dL were regarded to be signs of dyslipidemia (20). An HbA1c level of more than 6.5% and the use of glucose-lowering medications were both considered indicators of diabetes mellitus (21). Hypertension was identified as taking antihypertensive medications or an SBP of ≥140 mmHg and/or a DBP of ≥90 mmHg (22).

### 2.4 Outcome definitions

For the NHANES cohort, the “Fatty Liver Index” (FLI) developed as proof of the existence of NAFLD (23). Participants with FLI >60 were assumed to have NAFLD. The donor livers in the FAHSYU cohort were evaluated whether they were fatty liver or not through liver biopsy. In addition, the NAFLD fibrosis score (NFS) was sufficient to predict long-term outcomes like mortality and liver complications. NFS >0.676 in the context of NAFLD indicates that a participant has AHF. The overall survival information was also utilized to evaluate the outcomes of all participants in NHANES 1999–2018, and corresponding data were downloaded from the National Center for Health Statistics (NCHS). The deadline of follow-up for NHANES 1999–2018 was 31 December 2019.

### 2.5 Mendelian randomization

The two-sample Mendelian randomization (MR) analysis was conducted on the GWAS summary data on the IEU GWAS data portal (https://gwas.mrcieu.ac.uk/). The instrumental variables for glucose, albumin, and diabetes were extracted separately from ebi-a-GCST90025986 (24), ebi-a-GCST90018945 (25), and ebi-a-GCST90038633 (26). To reduce the bias from diabetes, multivariable MR was performed and the common instrumental variables with P-value<1e-5 were identified as significant single-nucleotide polymorphisms (SNPs). The summary-level data for NAFLD were extracted from finn-b-NAFLD (https://gwas.mrcieu.ac.uk/datasets/finn-b-NAFLD/). All these GWAS populations were European. The “TwoSampleMR” package was utilized to conduct the multivariable MR analysis. Then, the heterogeneity of this study was evaluated by MR-Egger and inverse variance-weighted (IVW) methods. Association strengths of genetic instruments for the above three risk factors were quantified by the F-statistic. Finally, we search the corresponding genes related to the significant SNPs in the National Center for Biotechnology Information (NCBI) database (https://www.ncbi.nlm.nih.gov/snp/).

### 2.6 Statistical analysis

Given the complex sampling design of NHANES, sampling weights were considered in the analysis process. We utilized 10 survey cycles to develop novel multiyear sample weights according to the analysis manual of NHANES. For the final analysis, we selected the weights of the smallest subpopulation that encompassed all variables.

In this research, normal distribution variables were described using the weighted mean (standard errors; SEs), whereas skewed distribution variables were exhibited by the median (interquartile range; IQR). Categorical variables were summarized using weighted frequency (standard errors, SEs). We used the Wilcoxon rank-sum test and chi-square test with Rao–Scott’s second-order correction to compare continuous and categorical variables, respectively. **Table 1** was created using the “gtsummary” package.

**Table 1:** Baseline characteristics of participants.

Characteristic	Normal (N = 40,661,234; 63.6%)	NAFLD (N = 23,246,927; 36.4%)	P
**Age** *(n, %)* **^★^**			0.998
Elders	22.6 (0.01)	22.6 (0.01)	
Young	77.4 (0.01)	77.4 (0.01)	
**Gender** *(n, %)* **^★^**			<0.001
Male	41.6 (0.01)	54.8 (0.01)	
Female	58.4 (0.01)	45.2 (0.01)	
**BMI** *(kg/m^2^)* **^▼^**	24.7 (0.05)	33.5 (0.10)	<0.001
**WC** *(cm)* **^▼^**	87.7 (0.14)	110.7 (0.21)	<0.001
**Race** *(n, %)* **^★^**			<0.001
Mexican American	6.6 (0.00)	8.0 (0.01)	
Non-Hispanic black	10.1 (0.01)	10.1 (0.01)	
Non-Hispanic white	70.0 (0.01)	72.3 (0.01)	
Other race	13.4 (0.01)	9.6 (0.01)	
**Education Level** *(n, %)* **^★^**			<0.001
College graduate or above	64.9 (0.01)	58.9 (0.01)	
High school or equivalent	21.1 (0.01)	24.9 (0.01)	
Less than 12th grade	14.0 (0.01)	16.2 (0.01)	
**Family PIR ^▲^**	3.4 [1.70, 5.00]	3.2 [1.62, 5.00]	0.006
**Smoking Status** *(n, %)* **^★^**			0.011
Current	15.9 (0.01)	16.6 (0.01)	
Ever	6.8 (0.00)	9.2 (0.01)	
Never	77.3 (0.01)	74.2 (0.01)	
**Leisure Activity** *(MET-min/day)* **^▲^**	150.7 [60.67, 329.39]	109.8 [45.00, 268.61]	<0.001
**Energy Intake** *(kcal/day)* **^▼^**	2,074.7 (10.15)	2,183.6 (15.46)	<0.001
**Protein Intake** *(g/day)* **^▼^**	79.0 (0.51)	84.8 (0.72)	<0.001
**Carbohydrate Intake** *(g/day)* **^▼^**	255.5 (1.39)	260.5 (2.05)	0.007
**Total fat Intake** *(g/day)* **^▼^**	78.9 (0.56)	86.3 (0.81)	<0.001
**Albumin** *(g/L)* **^▼^**	43.1 (0.06)	42.2 (0.07)	<0.001
**ALT** *(U/L)* **^▲^**	19.0 [15.00, 24.00]	25.0 [19.00, 34.00]	<0.001
**AST** *(U/L)* **^▲^**	22.0 [19.00, 25.00]	23.0 [19.17, 28.00]	<0.001
**ALP** *(U/L)* **^▲^**	62.0 [51.00, 75.00]	69.0 [57.00, 84.00]	<0.001
**GGT** *(U/L)* **^▲^**	16.0 [12.00, 21.00]	26.0 [19.00, 38.00]	<0.001
**Total bilirubin** *(µmol/L)* **^▲^**	12.0 [8.55, 15.39]	10.3 [8.55, 13.68]	<0.001
**Triglyceride** *(µmol/L)* **^▲^**	83.0 [61.00, 114.00]	146.7 [103.00, 205.00]	<0.001
**Total cholesterol** *(µmol/L)* **^▼^**	191.2 (0.59)	204.1 (0.88)	<0.001
**HDL cholesterol** *(µmol/L)* **^▼^**	59.0 (0.25)	46.8 (0.21)	<0.001
**LDL cholesterol** *(µmol/L)* **^▼^**	113.5 (0.51)	123.5 (0.69)	<0.001
**WBC** *(× 10^9/L)* **^▲^**	6.0 [5.10, 7.20]	6.9 [5.80, 8.10]	<0.001
**Lymphocyte** *(× 10^9/L)* **^▲^**	180.6 [148.20, 221.13]	199.5 [165.30, 241.30]	<0.001
**Monocyte** *(× 10^9/L)* **^▲^**	48.9 [39.65, 59.76]	53.1 [43.56, 64.60]	<0.001
**Neutrophils** *(× 10^9/L)* **^▲^**	342.2 [269.50, 438.07]	395.1 [315.51, 496.20]	<0.001
**Eosinophils** *(× 10^9/L)* **^▲^**	14.7 [9.43, 23.24]	17.6 [11.62, 26.24]	<0.001
**Basophils** *(× 10^9/L)* **^▲^**	3.9 [2.55, 5.58]	4.4 [2.95, 6.23]	<0.001
**RBC** *(× 10^9/L)* **^▼^**	4.7 (0.01)	4.9 (0.01)	<0.001
**PLT** *(× 10^9/L)* **^▼^**	246.0 (1.03)	260.5 (1.18)	<0.001
**HbA1c** *(%)* **^▲^**	5.3 [5.10, 5.50]	5.5 [5.20, 5.70]	<0.001
**Plasma glucose** *(mmol/L)* **^▲^**	5.3 [4.97, 5.66]	5.6 [5.27, 6.00]	<0.001
**Dyslipidemia** *(n, %)* **^★^**	40.5 (0.01)	74.1 (0.01)	<0.001
**Hypertension** *(n, %)* **^★^**	99.4 (0.00)	98.0 (0.00)	<0.001
**GAR ^▼^**	12.5 (0.03)	13.6 (0.04)	<0.001

Data are shown as the weighted mean (standard errors; SEs) ▼, median (interquartile range; IQR) ▲, or weighted frequency (standard errors; SEs)★ as appropriate. The Wilcoxon rank-sum test for continuous variables and the chi-square test with Rao–Scott’s second-order correction for categorical variables were used in this analysis.

BMI, body mass index; WC, waist circumference; Family PIR, family poverty income ratio; ALT, alanine aminotransferase; AST, aspartate aminotransferase; ALP, alkaline phosphatase; GGT, γ-glutamyl transferase; LDL cholesterol, low-density lipoprotein cholesterol; HDL cholesterol, high-density lipoprotein cholesterol; WBC, white blood cell; RBC, red blood cell; PLT, platelet; HbA1c, glycated hemoglobin; GAR, glucose-to-albumin ratio.

We used a univariate logistic regression analysis to investigate the factors linked to NAFLD in people without diabetes. Furthermore, to ascertain the association between GAR and NAFLD, we used a multivariate logistic regression analysis. Covariates included age, gender, hypertension, race, education level, family PIR, smoking status, leisure activity, energy, and dyslipidemia. The survival analysis was presented on the K-M plots to verify the poor prognosis of NAFLD, AHF, and GAR. In addition, the Pearson correlation analysis was utilized to investigate the relationship between GAR and liver fibrosis of NAFLD subjects. Statistical significance was determined at a threshold of p< 0.05 (two-sided). These analyses were conducted in R 4.2.0 using the “survey” package.

## 3 Results

### 3.1 The baseline characteristics of participants in the NHANES cohort

A total of 15,534 eligible participants were included in this research. The characteristics were compared between normal and NAFLD patients without AHF through weighting analysis (**Table 1**). It was obvious that women tend to be at high risk of NAFLD. There was a significant difference in the contour of body, economic level, education background, living habit, and dietary habits. NAFLD patients appeared to have a higher BMI and WC. The participants with lower family PIR and education level present a high prevalence of NAFLD, and these patients were combined with some unhealthy habits like smoking and lower leisure activity. In addition, NAFLD patients were accustomed to high energy, protein, carbohydrate, and fat intake. Therefore, serum triglyceride, total cholesterol, and LDL cholesterol accumulated to a higher level in NAFLD patients, while HDL cholesterol decreased. Aside from these clinical characteristics, there were higher white blood cell count (lymphocyte, monocyte, neutrophils, eosinophils, and basophils), red blood cell count, and platelet count in the peripheral blood sample from NAFLD patients. Serum albumin was lower in NAFLD patients, while liver enzymes (ALT, AST, and ALP) were higher than those in the normal patients. The indicators of biliary obstruction (GGT) were higher in the NAFLD group. The metabolism syndrome was verified to be related to the prevalence of NAFLD, with higher glycohemoglobin, plasma glucose, high risk of dyslipidemia, and lower prevalence of hypertension in the NAFLD group. Most interestingly, the GAR presented a higher ratio in NAFLD patients.

### 3.2 GAR was associated with the prevalence of NAFLD

**Figure 2:**
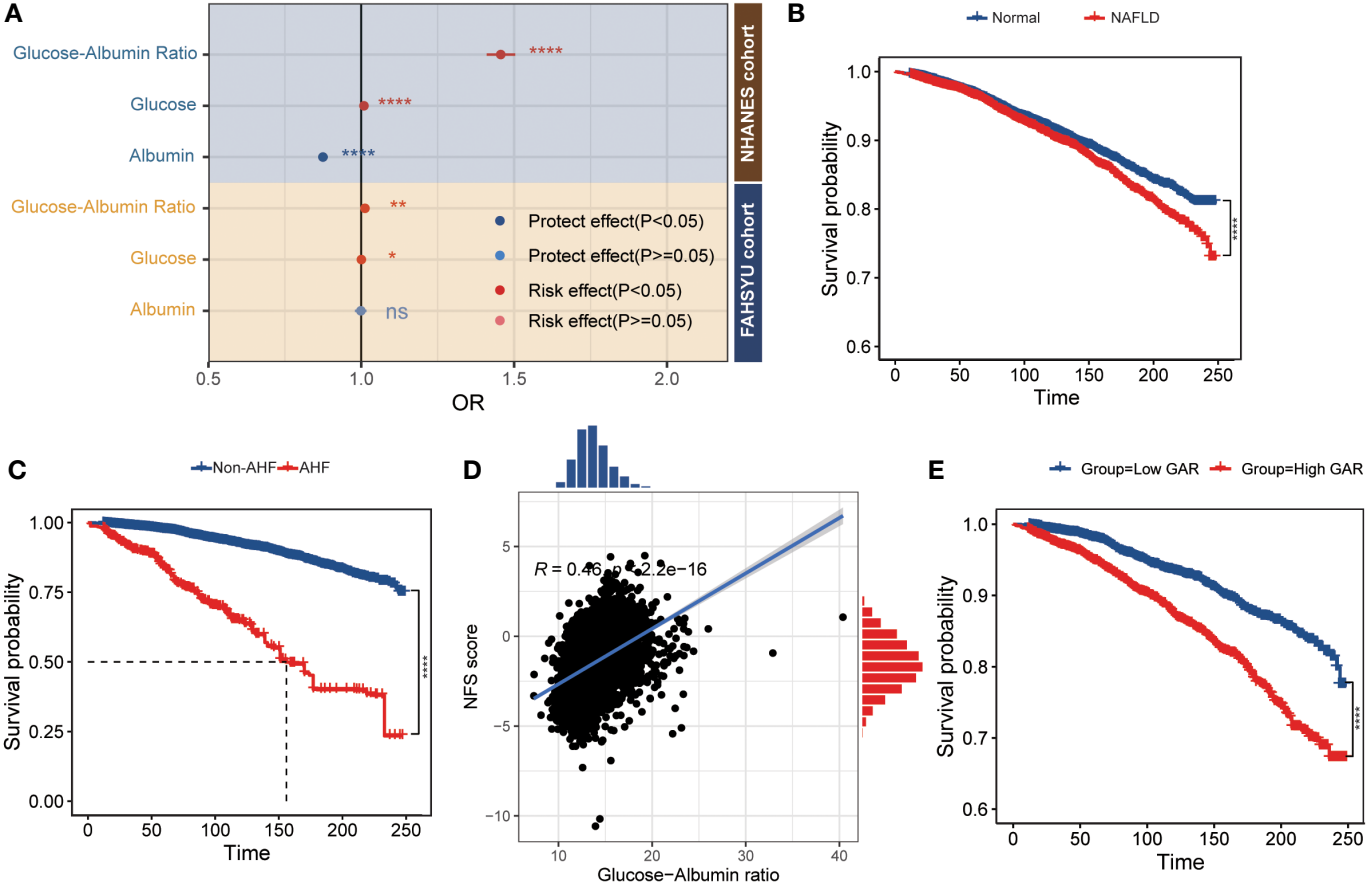
The relationship between GAR and prognosis of NAFLD patients. **(A)** The forest plot comparing the odds ratio for NAFLD among glucose, albumin, and GAR [adjust age (elders and young), gender (male and female), and hypertension]. **(B)** The survival plot of normal and NAFLD patients. **(C)** The survival plot of non-AFL and AFL patients. **(D)** The Pearson correlation plot between GAR and NFS score. **(E)** The K-M plot of GAR in NAFLD patients (grouped by the median of GAR) *P < 0.05, **P < 0.01, ***P < 0.001, ****P < 0.0001. NHANES, National Health and Nutrition Examination Survey; FAHSYU, First Affiliated Hospital of Sun Yat-sen University; NAFLD, nonalcoholic fatty liver disease; AHF, advanced hepatic fibrosis; NFS, NAFLD fibrosis score; GAR, glucose-to-albumin ratio.

It can be inferred that GAR acts as a risk factor in the development of NAFLD. Among patients without AHF, the multivariable logistic analysis was introduced and the corresponding result was presented in **Table 2** and **Supplementary Table S2**. After multivariable adjustment for some known and/or potential covariates, GAR was positively related to the prevalence of NAFLD (model 1: OR = 1.46, 95% CI 1.41–1.50, P< 0.001; model 2: OR = 1.56, 95% CI 1.43–1.71, P< 0.001; model 3: OR = 1.53, 95% CI 1.39–1.67, P< 0.001). In addition, model 1 was verified in the FAHSYU cohort (OR = 1.01, 95% CI 1.00–1.02, P = 0.003, **Supplementary Table S2**). Taken together, it was obvious that GAR was demonstrated as an independent risk factor for NAFLD in both the NHANES cohort and the FAHSYU cohort. Except for the GAR, other covariates (age, gender, hypertension, leisure activity, and dyslipidemia) could be concluded as an independent risk or protective factor in the progress of NAFLD. In more detail, female gender and leisure activity played important roles in the prevention of NAFLD, while others were promoters. In addition, through comparing the GAR, glucose, and albumin in both NHANES cohort and FAHSYU cohort, it was summarized that GAR seemed to be a more efficient, accurate, and important risk factor in the development of NAFLD (**Figure 2A**). Glucose seemed to be a risk promoter for NAFLD, while there was a protective role for albumin.

**Table 2:** The correlation between GAR and risk of NAFLD.

Characteristic	Model 1	P	Model 2	P	Model 3	P
	OR (95% CI)		OR (95% CI)		OR (95% CI)	
**GAR**	1.46 (1.41- 1.50)	<0.001	1.56 (1.43- 1.71)	<0.001 5	1.53 (1.39- 1.67)	<0.001
Age						
Elders	—		—		—	
Young	1.45 (1.28- 1.63)	<0.001	1.43 (1.08- 1.88)	0.013	1.71 (1.29- 2.28)	<0.001
Gender						
Male	—		—		—	
Female	0.56 (0.51- 0.62)	<0.001	0.41 (0.32-0.52)	<0.001	0.49 (0.38- 0.63)	<0.001
Hypertension						
No	—		—		—	
Yes	0.37 (0.24-0.56)	<0.001	0.25 (0.11-0.56)	0.001	0.22 (0.08- 0.56)	0.002
Race						
Mexican American			—		—	
Non-Hispanic black			0.89 (0.58- 1.36)	0.6	0.99 (0.63- 1.55)	>0.9
Non-Hispanic white			0.83 (0.59- 1.18)	0.3	0.74 (0.52- 1.05)	0.091
Other race			0.73 (0.46- 1.16)	0.2	0.59 (0.36- 0.97)	0.036
Education level						
College graduate or above			—		—	
High school or equivalent			1.26 (0.96- 1.64)	0.089	1.15 (0.88- 1.50)	0.3
Less than 12th grade			0.91 (0.63- 1.36)	0.7	0.92 (0.63- 1.34)	0.7
**Family PIR**			0.98 (0.91-1.06)	0.7	0.98 (0.90- 1.06)	0.6
Smoking status						
Current			—		—	
Ever			1.34 (0.76- 2.35)	0.3	1.15 (0.68- 1.94)	0.6
Never			1.09 (0.71- 1.68)	0.7	1.06 (0.70-1.60)	0.8
**Leisure activity**			1.00 (1.00- 1.00)	<0.001	1.00 (1.00- 1.00)	<0.001
**Energy intake**			1.00 (1.00- 1.00)	0.3	1.00 (1.00- 1.00)	0.3
Dyslipidemia						
No					—	
Yes					4.50 (3.49- 5.80)	<0.001

Model 1: age (elders and young), gender (male and female), hypertension. Model 2: model 1 + race (Mexican American, other Hispanic, non-Hispanic white, non-Hispanic black, other race), education level (less than 12th grade, high school or equivalent, college graduate or above), family PIR, smoking status, leisure activity (MET-min/day), and energy intake (kcal/day). Model 3: model 2 + dyslipidemia. GAR, glucose-to-albumin ratio; WC, waist circumference; Family PIR, family poverty income ratio.

### 3.3 GAR was related to the liver fibrosis and mortality among NAFLD patients

Consistent with the former result that the liver function-related markers like AST, ALT, ALP, and GGT were upregulated in NAFLD patients (**Table 1**), there was a poor prognosis in NAFLD patient (**Figure 2B**). Among the NAFLD patients, AHF was associated with a poor survival (**Figure 2C**). Through comparing the different clinical characteristics between NAFLD with or without fibrosis, it was demonstrated that an elderly woman with higher BMI, WC, energy, protein, and carbohydrate intake was more likely to develop AHF (**Table 3**). Most importantly, AHF patients were accomplished with a higher GAR, which was consistent with the correlation analysis between GAR and NFS (**Figure 2D**). To further investigate the relationship between GAR and the prognosis of NAFLD, the survival analysis was carried out among NAFLD patients, and it was obvious that NAFLD patients with higher GAR tended to suffer a poor survival (**Figure 2E**).

**Table 3:** Different characteristics between NAFLD patients with and without fibrosis.

Characteristic	NAFLD without fibrosis (N = 23,246,927; 93.8%)	NAFLD with fibrosis (N = 1,540,710; 6.2%)	P
**Age** *(n, %) ★*			<0.001
Elders	22.6 (0.01)	74.3 (0.03)	
Young	77.4 (0.01)	25.7 (0.03)	
**Gender** *(n, %) ★*			0.055
Male	54.8 (0.01)	47.8 (0.04)	
Female	45.2 (0.01)	52.2 (0.04)	
**BMI** *(kg/m^2^) ▼*	33.5 (0.10)	39.0 (0.57)	<0.001
**WC** *(cm) ▼*	110.7 (0.21)	122.8 (1.05)	<0.001
**Race** *(n, %) ★*			0.169
Mexican American	8.0 (0.01)	5.2 (0.01)	
Non-Hispanic black	10.1 (0.01)	10.4 (0.01)	
Non-Hispanic white	72.3 (0.01)	75.4 (0.03)	
Other race	9.6 (0.01)	9.1 (0.01)	
**Education Level** *(n, %) ★*			0.670
College graduate or above	58.9 (0.01)	56.3 (0.03)	
High school or equivalent	24.9 (0.01)	26.3 (0.03)	
Less than 12th grade	16.2 (0.01)	17.4 (0.02)	
**Family PIR** *▲*	3.2 [1.62, 5.00]	2.5 [1.45, 4.89]	0.099
**Smoking Status** *(n, %) ★*			0.062
Current	16.6 (0.01)	9.0 (0.02)	
Ever	9.2 (0.01)	10.0 (0.02)	
Never	74.2 (0.01)	81.0 (0.03)	
**Leisure activity** *(MET-min/day) ▲*	109.8 [45.00, 268.61]	84.2 [29.42, 141.84]	0.078
**Energy intake** *(kcal/day) ▼*	2,183.6 (15.46)	2,019.9 (48.60)	0.006
**Protein intake** *(g/day) ▼*	84.8 (0.72)	77.8 (2.46)	0.012
**Carbohydrate intake** *(g/day) ▼*	260.5 (2.05)	232.4 (5.20)	<0.001
**Total fat intake** *(g/day) ▼*	86.3 (0.81)	84.6 (3.12)	0.488
**Albumin** *(g/L) ▼*	42.2 (0.07)	40.3 (0.23)	<0.001
**ALT** *(U/L) ▲*	25.0 [19.00, 34.00]	20.0 [15.00, 25.67]	<0.001
**AST** *(U/L) ▲*	23.0 [19.17, 28.00]	24.0 [20.00, 27.00]	0.542
**ALP** *(U/L) ▲*	69.0 [57.00, 84.00]	70.0 [56.00, 85.00]	0.970
**GGT** *(U/L) ▲*	26.0 [19.00, 38.00]	22.0 [17.00, 33.00]	<0.001
**Total Bilirubin** *(µmol/L) ▲*	10.3 [8.55, 13.68]	10.3 [8.55, 13.68]	0.481
**Triglyceride** *(µmol/L) ▲*	146.7 [103.00, 205.00]	128.0 [92.00, 167.00]	<0.001
**Total Cholesterol** *(µmol/L) ▼*	204.1 (0.88)	185.1 (2.54)	<0.001
**HDL Cholesterol** *(µmol/L) ▼*	46.8 (0.21)	50.5 (1.04)	<0.001
**LDL Cholesterol** *(µmol/L) ▼*	123.5 (0.69)	107.1 (2.33)	<0.001
**WBC** *(× 10^9/L) ▲*	6.9 [5.80, 8.10]	6.6 [5.50, 7.70]	0.016
**Lymphocyte** *(× 10^9/L) ▲*	199.5 [165.30, 241.30]	180.1 [136.20, 215.46]	<0.001
**Monocyte** *(× 10^9/L) ▲*	53.1 [43.56, 64.60]	54.1 [44.39, 65.61]	0.278
**Neutrophils** *(× 10^9/L) ▲*	395.0 [315.51, 496.20]	395.4 [307.48, 479.53]	0.347
**Eosinophils** *(× 10^9/L) ▲*	17.6 [11.62, 26.24]	18.7 [13.09, 26.22]	0.117
**Basophils** *(× 10^9/L) ▲*	4.4 [2.95, 6.23]	4.6 [3.04, 6.24]	0.734
**RBC** *(× 10^9/L) ▲*	4.9 (0.01)	4.6 (0.03)	<0.001
**PLT** *(× 10^9/L) ▲*	260.5 (1.18)	193.1 (3.06)	<0.001
**Plasma Glucose** *(mmol/L) ▲*	5.6 [5.27, 6.00]	6.3 [6.05, 6.61]	<0.001
**Dyslipidemia** *(n, %) ★*	74.1 (0.01)	65.9 (0.03)	0.009
**Hypertension** *(mmol/L) ▲*	98.0 (0.00)	99.5 (0.00)	0.011
**GAR** *▼*	13.6 (0.04)	15.6 (0.11)	<0.001

Data are shown as the weighted mean (standard errors; SEs) ▼, median (interquartile range; IQR) ▲, or weighted frequency (standard errors; SEs) ★ as appropriate. The Wilcoxon rank-sum test for continuous variables and the chi-square test with Rao–Scott’s second-order correction for categorical variables were used in this analysis.

BMI, body mass index; WC, waist circumference; family PIR, family poverty income ratio; ALT, alanine aminotransferase; AST, aspartate aminotransferase; ALP, alkaline phosphatase; GGT, γ-glutamyl transferase; LDL cholesterol, low-density lipoprotein cholesterol; HDL cholesterol, high-density lipoprotein cholesterol; WBC, white blood cell; RBC, red blood cell; PLT, platelet; HbA1c, glycated hemoglobin; GAR, glucose-to-albumin ratio.

### 3.4 MR analysis for serum glucose and albumin

All F-statistics of the three exposure factors were over 10. The P-value of the heterogeneity test was both over 0.05, which implied that there was no heterogeneity in our research. Genetic predisposition to glucose [OR (95% CI): 8.50 (1.04–69.27), P = 0.046] was significantly associated with an increased risk of NAFLD (**Supplementary Table S3**). While there was a reverse trend for albumin [OR (95% CI): 0.42 (0.00–63.98), P = 0.736] (**Supplementary Table S3**). The significant instrument SNPs for glucose were rs10830963 (MTNR1B), rs11708067 (ADCY5), rs12454712 (BCL2), rs1359790 (LOC105370275), rs3802177 (SLC30A8), and rs7903146 (TCF7L2). The included SNPs for albumin were rs4805881 (PEPD), rs76895963 (CCND2), and rs79687284 (PROX1-AS1).

## 4 Discussion

The increasing prevalence of NAFLD made the fatty liver become more and more common among both worldwide population and donor pool (1–3). Several studies had developed some methods for noninvasive estimation of hepatic steatosis based on ultrasound or computed tomography image (27–31). However, the shortcomings of liver biopsy and imaging tests inspired us to develop more efficient clinical prediction tools. Both plasma glucose and albumin reflected the protein metabolism and glycometabolism of liver and even the whole body. GAR was revealed as a risk factor for spontaneous intracerebral hemorrhage (14). Therefore, we investigated the potential prognostic value of GAR in NAFLD.

In this cross-sectional research, the baseline characteristics between normal subjects and NAFLD patients were compared to enhance the reliability of the study. Men who are overweight and have smoked in the past are considerably more likely to have NAFLD, according to a systematic review and meta-analysis of 63 studies involving 1,201,807 individuals (1), which was consistent with our result that NAFLD patients appeared to have a higher male ratio, BMI/WC, and smoking ratio. In addition, we assumed that the higher BMI for NAFLD patients attributed to the higher energy intake including protein, carbohydrate, and fat, which also promoted the elevation of blood lipid level and finally the development of dyslipidemia. The multivariable logistic regression analysis indicated that GAR is a distinct risk factor for NAFLD. In addition, the FAHSYU cohort not only validated the positive relationship between GAR and fatty liver but also extended the population applicability of GAR to liver donors. Additionally, GAR and liver fibrosis in NAFLD patients showed a favorable association, even with mortality. To further verify the cause-and-effect between and GAR and NAFLD, MR analysis revealed that higher serum glucose concentration led to a high prevalence of NAFLD while that of albumin was reversed. In our research, we insisted that GAR representing both protein metabolism and glycometabolism contributed to the development of NAFLD and even liver fibrosis.

A great number of research had demonstrated that serum glucose could be responsible for the development and progress of NAFLD. The “two-hit” hypothesis was illustrated by Day and James to explain the development of NAFLD (32). Hepatic steatosis development is the initial strike, then hepatocyte mortality results from oxidative, metabolic, and cytokine stressors that overwhelm hepatocyte survival systems. Additionally, hepatocyte apoptosis is regarded by some researchers as a third driver that accelerates the development of cirrhosis (33). Several studies demonstrated that glucose led to the hepatic de novo lipogenesis (DNL) through activating carbohydrate-responsive element-binding protein (ChREBP) (34, 35), a glucose sensor. Régnier et al. (36) assumed that ChREBP induces liver fat accumulation without leading to insulin resistance, which was consistent with the risk role of GAR in adults without diabetes. In addition, it was demonstrated that glucose fluctuation contributed to the hepatic apoptosis, inflammation, and fibrosis through elevated oxidative stress both *in vivo* and *in vitro* (9, 10). Therefore, glucose was included in several combinatorial indexes to identify the early pathophysiological state of NAFLD like TyG, TyG-WC, TyG-WHtR, and TyG-BMI (11–13). The above information implied that a single factor like glucose was not sufficient for the prediction of NAFLD.

Serum albumin, another possible predictive factor, was thought to represent a new biomarker of early liver function decline (37). The antioxidant and anti-inflammatory function of albumin implied its potential prognostic value for NAFLD. The fatty acid oxidation process generates reactive oxygen species (38), mediates oxidative stress, and then damages the ultrastructure and antioxidant system of mitochondria (glutathione, glutathione peroxidase, and superoxide dismutase), forming an irreversible vicious cycle. In addition, reactive oxygen species, as the cornerstone of NAFLD development, also participate in the regulation of inflammation (39). Large amounts of reactive oxygen species in liver tissues further affect local inflammation and immunity, including both innate and acquired immunity (39). It was demonstrated that plasma albumin level decreased in NAFLD patients (40). Therefore, albumin also shows an inspiring prospect in predicting the prevalence of NAFLD.

Through the MR analysis, we investigated the underlying genetic cause-and-effect between GAR and NAFLD. The trend of OR value of both glucose and albumin implied the risk role of GAR in adults without diabetes for NAFLD, although the P-value for albumin was not significant due to the limitation of online GWAS profiles. In addition, decreased albumin level also reflects worse liver function, which might imply more chances of metabolic disorder in liver cells. As a result, GAR could function as a glucose level adjusted with albumin for the prediction of NAFLD. Through MR analysis, we identified several genetic relationships between GAR and NAFLD like MTNR1B, ADCY5, BCL2, LOC105370275, SLC30A8, TCF7L2, PEPD, CCND2, and PROX1-AS1. BCL2 functioned as an antiapoptosis gene to alleviate liver damage in NAFLD. Adaptive upregulation of BCL2 was determined in NAFLD (41), while there was a significant low level of BCL2 and elevated apoptosis in nonalcoholic steatohepatitis (NASH) (42). An acridone derivative A22 could upregulate the expression of BCL2, which will reduce apoptosis, improve lipid and glucose metabolism in NAFLD (43). The polymorphism of TCF7L2 was determined as a risk factor for NAFLD and NASH (44, 45). And it could affect the lipid and glucose metabolism for NASH (45). TCF7L2 could contribute to the DNL of mouse hepatic cells through utilization of excess serum glucose (46). Therefore, our analysis presented the potential therapy targets for high GAR patients.

There were two main advantages of this study: the great number of participants and the validation NAFLD cohort based on liver biopsy. However, there were still several limitations in our research. First, due to the missing value of insulin levels, a more thorough multivariate analysis needed to be conducted in large population cohorts. In addition, only several important confounding factors were adjusted in the FAHSYU cohort with limited parameter information, which implied that two analyses could be only partially compared. Second, due to little information in the NHANES dataset about the diagnosis of cirrhosis, liver cirrhosis was identified by AHF. Furthermore, our cohort for validation might contain some donors with alcoholic fatty liver disease due to the missing information about alcoholic consumption. Therefore, further research was necessary to determine the clinical application value and underling molecular mechanism.

## 5 Conclusions

In summary, the distinguished clinical characteristics of NAFLD, including higher male ratio, BMI/WC, smoking ratio, energy intake, blood lipid level, white blood cell amount, and lower leisure activity, were verified in our research. Moreover, the relationship between NAFLD and metabolism syndromes (diabetes, dyslipidemia, and hypertension) was consistent with that of former studies (4, 5, 47). Most importantly, this study provided a unique early prognostic marker for NAFLD, which could further predict the liver fibrosis and long-time outcomes. In addition, two genetic therapy targets (BCL2 and TCF7L2) were identified for higher GAR in NAFLD patients.

## Data availability statement

The raw data supporting the conclusions of this article will be made available by the authors, without undue reservation.

## Ethics statement

The studies involving humans were approved by the Institutional Ethics Committee for Clinical Research and Animal Trials of the First Affiliated Hospital of Sun Yat-sen University. The studies were conducted in accordance with the local legislation and institutional requirements. The ethics committee/institutional review board waived the requirement of written informed consent for participation from the participants or the participants’ legal guardians/next of kin because the retrospective, minimal-risk nature of the study.

## Author contributions

SW: Conceptualization, Formal analysis, Methodology, Software, Visualization, Writing – original draft. XL: Data curation, Writing – original draft. CZ: Data curation, Writing – original draft. YD: Writing – review & editing. YG: Writing – review & editing. ZX: Writing – review & editing. XH: Supervision, Writing – review & editing. WJ: Supervision, Writing – review & editing. MC: Supervision, Writing – review & editing.

## Supplementary material

Supplementary Table 1 The cause of death for all donors.

Supplementary Table 2 Validation of the relationship between the GAR and the prevalence of FLD. *GAR* Glucose-Albumin Ratio. *BMI* body mass index.

Supplementary Table 3 Genetically predicted diabetes-adjusted associations of genetically predicted glucose and albumin with risk of nonalcoholic fatty liver disease in the combined datasets. *CI* confidence interval, *OR* odds ratio, *IVW* inverse-variance weighted.
