# Supplementary material for: Association between nonalcoholic fatty liver disease and increased glucose-to-albumin ratio in adults without diabetes
Source: Front Endocrinol (Lausanne). 2024 Jan 8;14:1287916. doi: 10.3389/fendo.2023.1287916 (PMC10804880; doi:10.3389/fendo.2023.1287916)
Supplement: Supplementary Table 1 — The cause of death for all donors. [file Table_1.docx]

**Table S1.** The cause of death for all donors.

| **Cause of Death** | **Proportion (n, %)** |
| --- | --- |
| Cerebral Vascular Accident | 199 (45.2%) |
| Severe Trauma | 194 (44.2%) |
| Hypoxic Ischemic Encephalopathy | 38 (8.6%) |
| Cerebral Tumor | 5 (1.1%) |
| Central Nervous System Infection | 4 (0.9%) |

**Table S2.** Validation of the relationship between the GAR and the prevalence of FLD

| **Characteristic** | **OR** | **95% CI** | **p-value** |
| --- | --- | --- | --- |
| **GAR** | 1.01 | 1.00, 1.02 | 0.003 |
| **Age** |  |  |  |
| Elders | — | — |  |
| Youngs | 0.65 | 0.24, 1.82 | 0.4 |
| **Gender** |  |  |  |
| Female | — | — |  |
| Male | 0.72 | 0.46, 1.12 | 0.14 |
| **Hypertension** | 2.14 | 0.81, 5.80 | 0.12 |

*GAR* Glucose-Albumin Ratio. *BMI* body mass index.

**Table S3**. Genetically predicted diabetes-adjusted associations of genetically predicted glucose and albumin with risk of nonalcoholic fatty liver disease in the combined datasets.

| **Exposure** | **OR (95%CI)** | **P value** | **MR-Egger** | **IVW** | **F-statistics** |
| --- | --- | --- | --- | --- | --- |
| Glucose | 8.50 (1.04-69.27) | 0.046 | 0.2684 | 0.334 | 47.97011 |
| Albumin | 0.42(0.00-63.98) | 0.736 | NA | 0.431 | 11.63585 |

*CI* confidence interval, *OR* odds ratio, *IVW* inverse-variance weighted.
